# Supplementary material for: Free Energy Differences and Coexistence of Clathrate Structures II and H via Lattice-Switch Monte Carlo
Source: J Chem Theory Comput. 2026 Aug 5;22(16):8156–69. doi: 10.1021/acs.jctc.6c00748 (PMC13523642; doi:10.1021/acs.jctc.6c00748)
Supplement: Supplementary file 1 [file ct6c00748_si_001.pdf]

# Supporting Information for Publication

# Free energy differences and coexistence of clathrate structures II and H via lattice-switch Monte Carlo

Olivia S. Moro[ab], Nigel B. Wilding[b] and Vincent Ballenegger[a].

[a] *Université Marie et Louis Pasteur, CNRS, Institut UTINAM (UMR 6213), F-25000 Besançon, France*
[b] *HH Wills Physics Laboratory, Royal Fort, University of Bristol, Bristol BS8 1TL, United Kingdom*

The simulation input files of our production Lattice-Switch Monte Carlo simulations of empty and filled methane hydrates using the simulation program DL_MONTE are available at `https://pubs.acs.org/doi/suppl/10.1021/acs.jctc.6c00748/suppl_file/ct6c00748_si_002.zip` (file `Simulation_input_files.zip`). On an Apple MacBook Pro M1 laptop (produced in 2021), these simulations take about 1 hour to compute $10^7$ MC steps, and hence about two days to complete a full production run on a single processor.

Snapshots of the simulation boxes of empty clathrates with structures II and H are shown in Fig. S1. The simulations were performed at the temperature 294.15K (number rounded to 294 K in the text of the article).

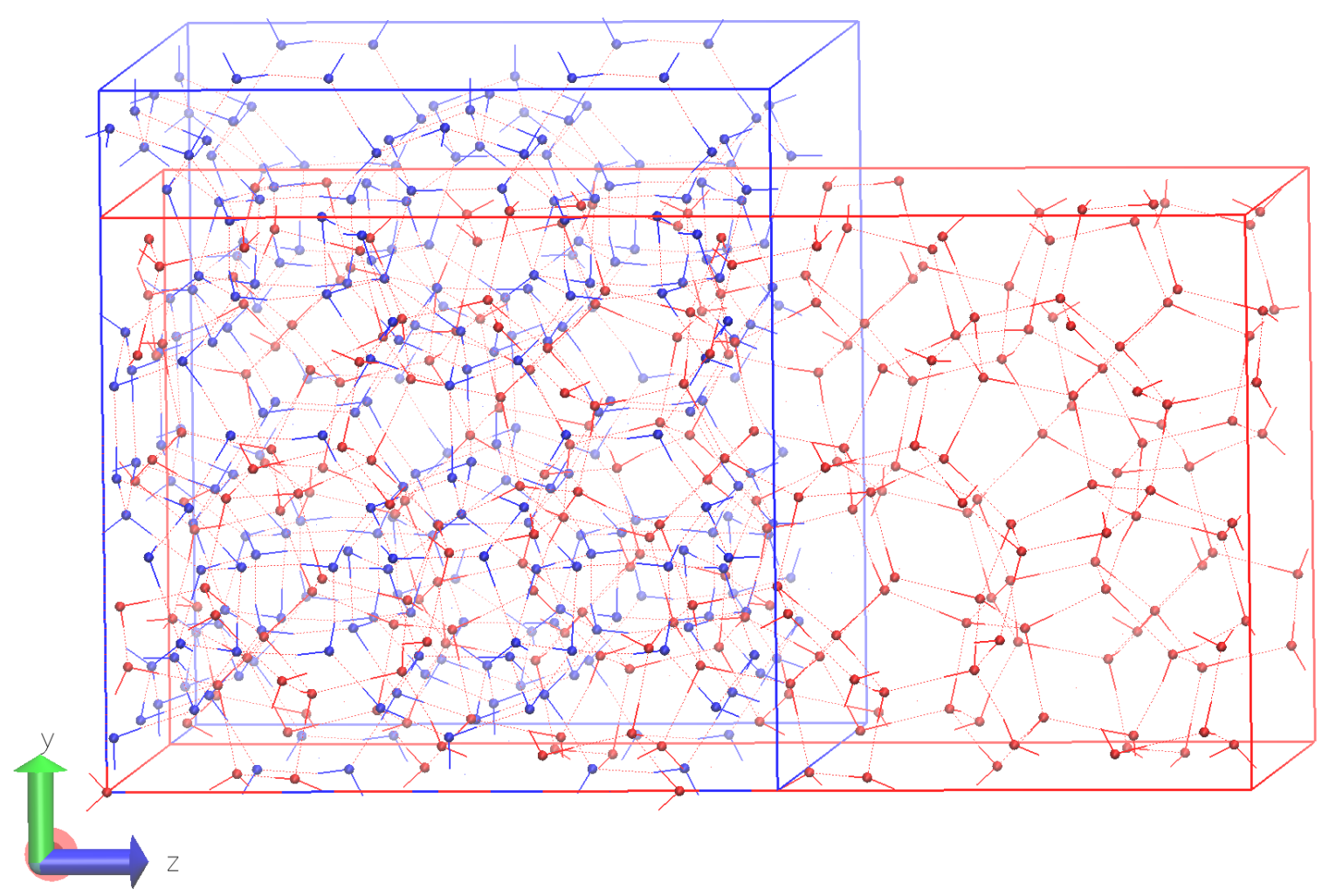


Figure S1: Two unit cells of an empty clathrate structure II (red orthorhombic box) alongside 8 unit cells with structure H (blue orthorhombic box). The blue box is almost cubic with cell dimensions $L_x = 2.442$, $L_y = 2.115$ and $L_z = 2.028$ nm. Each box contains 272 water molecules. Thin dashed lines represent hydrogen bonds.

# 1 Validation of the forcefield

The reliability of the numerical coexistence predictions is limited by the underlying intermolecular potentials. The choice of the TIP4P/Ice forcefield, together with standard Lennard-Jones interaction parameters for Ar and CH4, described as a united atom, is common in the literature. The phase diagram of the TIP4P/Ice water model has been determined up to high pressures[1, 2]. Gas hydrates have also been simulated up to high pressures (∼1 GPa) using TIP4P/Ice, see e.g. Ref. [3] and the justification given therein for selecting TIP4P/Ice. Fig. 3 of Ref. [3] shows the lattice constant of a hydrogen hydrate computed with TIP4P/Ice up to 1 Ga, alongside an experimental point.

A sI methane hydrate has been simulated in Ref. [4] with the same forcefield as in our calculations. The obtained structure is compared with experiments up to pressure 1000 bar. At 0.1 GPa (1000 bar), the relative error on the lattice constant is less than 1%. The TIP4P/Ice model overestimates somewhat the lattice constant (see Fig. 2 in Ref. [4]).

The pressure dependence of the unit cell volume of argon clathrates (see Fig. 9 of the main article) can be compared with the experiments of Manakov et al. [5] as their experimental setup corresponds well to our calculations (gas hydrate always in contact with excess argon gas when varying the pressure). Manakov et al. proposed parameters for Birch-Murnaghan fits to experimental data. The comparison shown in Fig. S2 indicates that our simulations capture the experimental trends with reasonable accuracy.

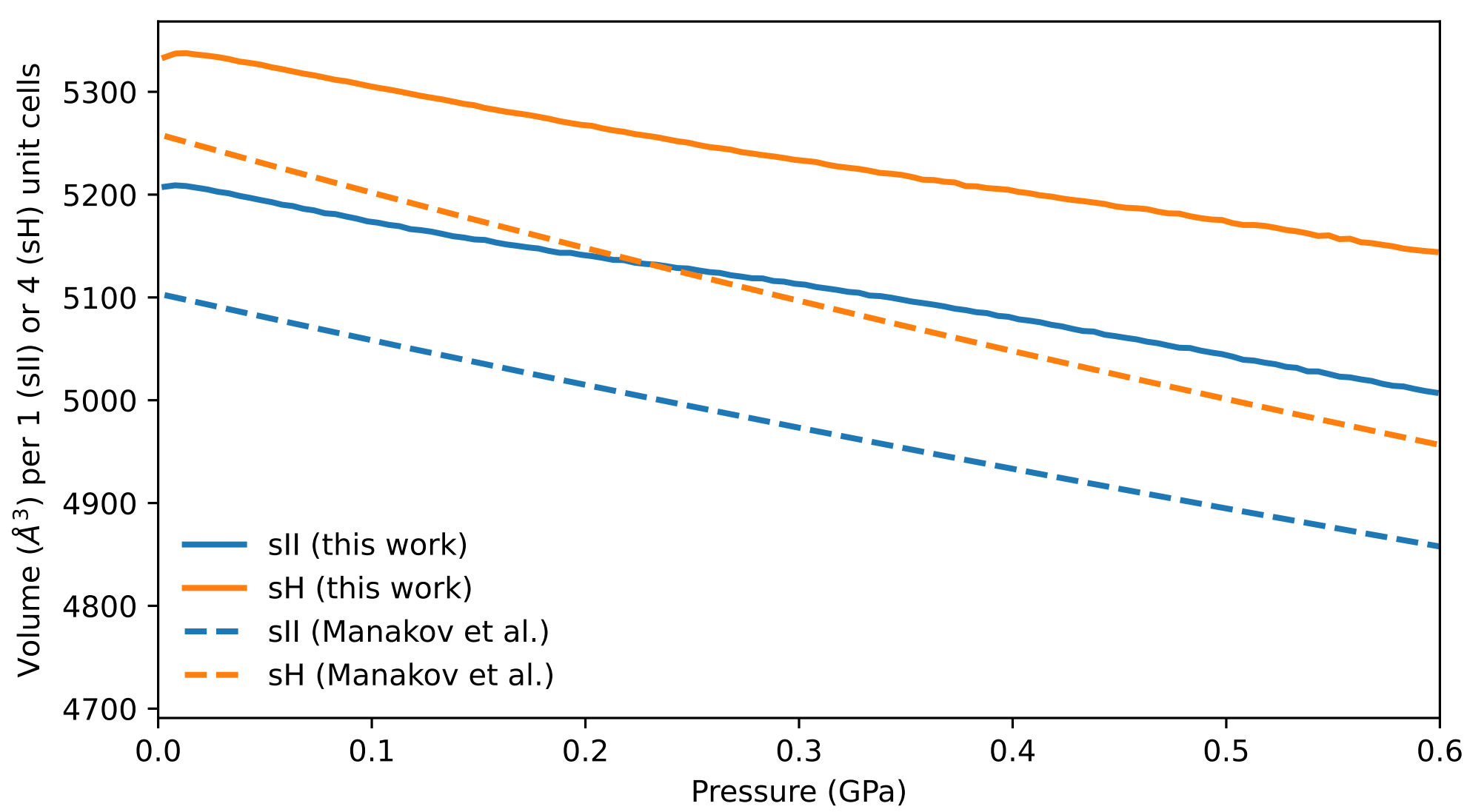


Figure S2: Volume of a structure II unit cell (blue line), and of 4 sH unit cells (orange line), as a function of pressure in our simulations compared to Birch-Murnaghan equations of state based on experimental data proposed by Manakov et al. [5] (dashed lines).

## 2 Additional plots

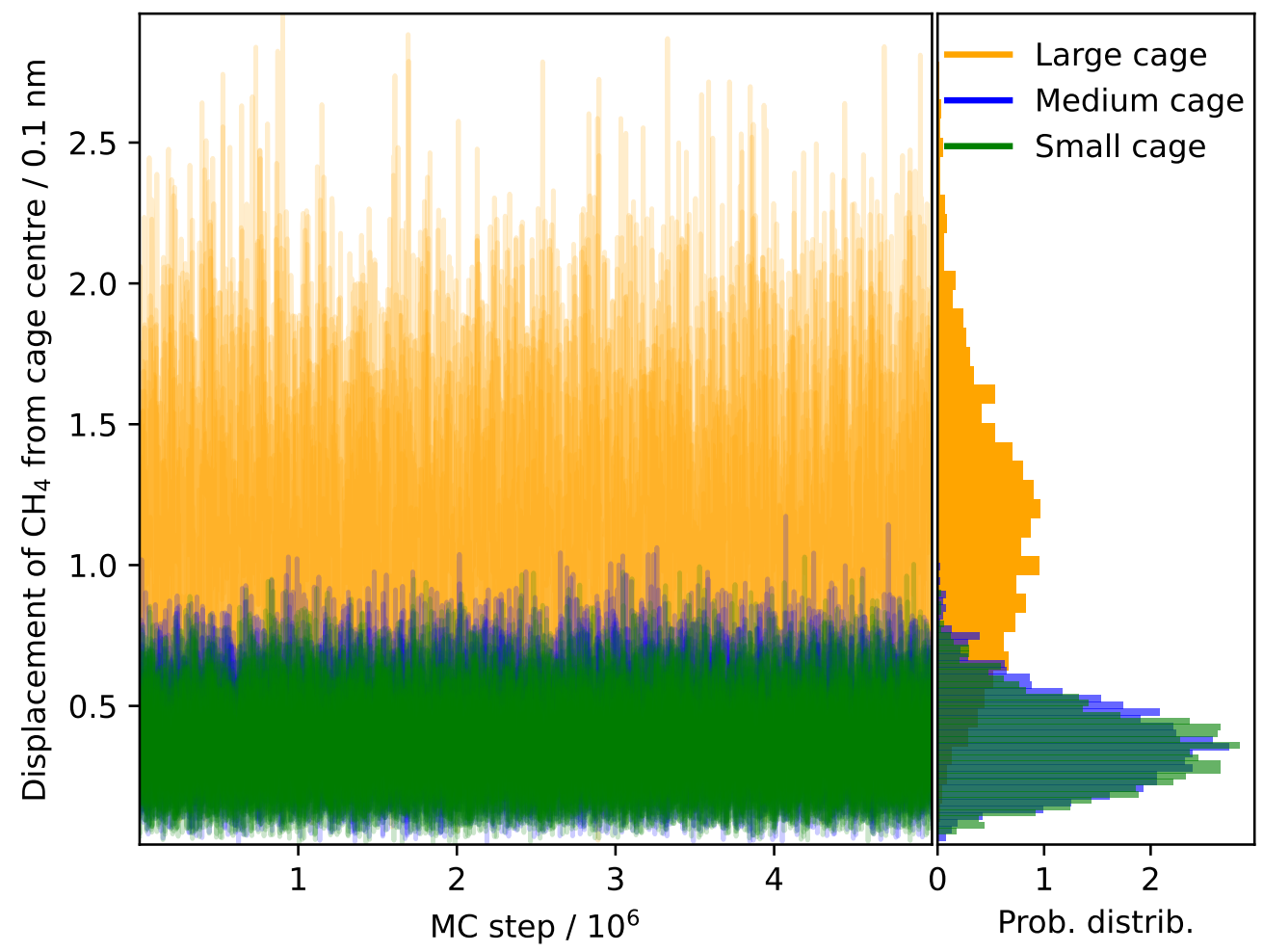


Figure S3: Norm of the displacement of each guest molecule from the center of its cage in a fully occupied methane sH clathrate at 30 bar and 294 K. The right panel shows the corresponding normalised histograms. Molecules are rarely found very close to the cage centre, partly due to a geometric effect: the volume element $4\pi r^2 \, dr$ decreases as $r \to 0$. No cage hopping is observed on the simulated timescale.

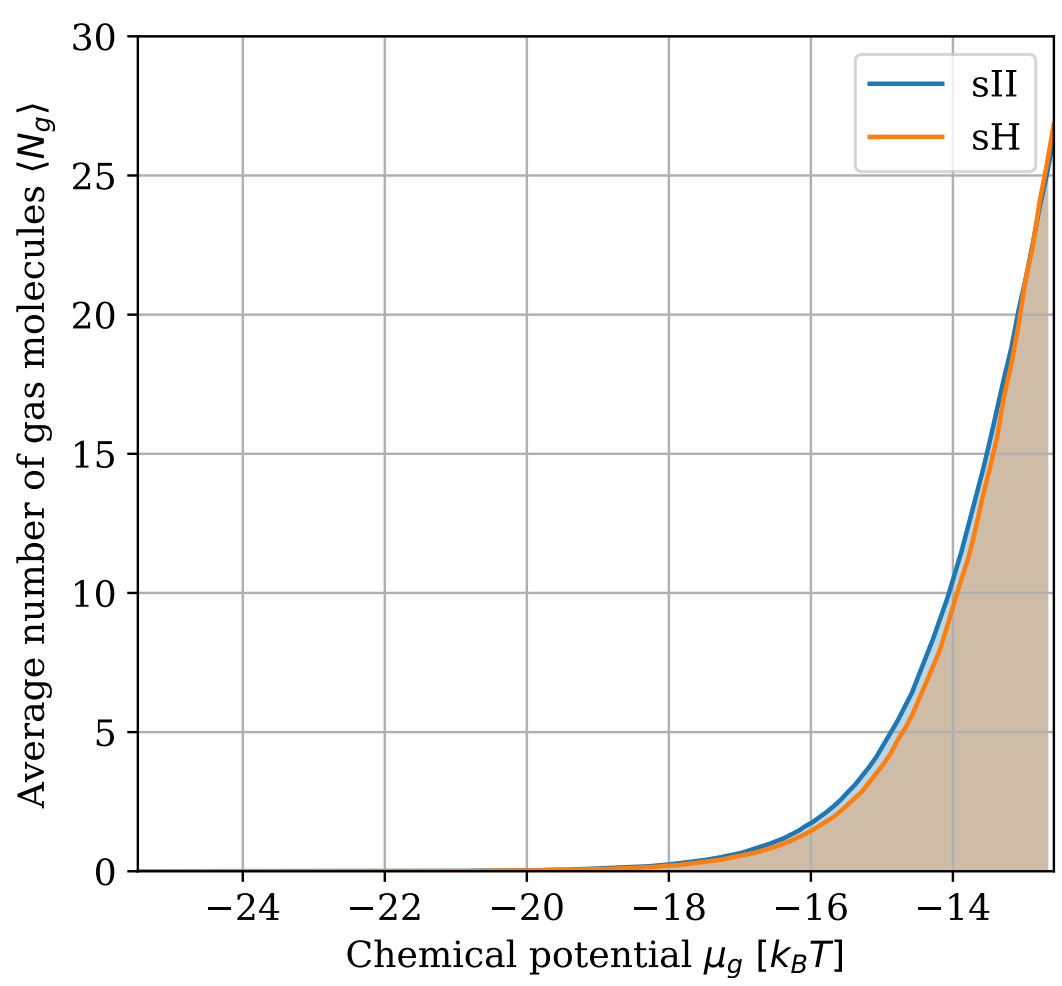


Figure S4: Average number of argon atoms in a hydrate with structure $\alpha =$ II or H as a function of the gas chemical potential (mechanical stress $P = 30$ bar, $T = 294$ K). The number of cages is 48. The shaded area shows the free energy $\Delta\Gamma^{\alpha}_{\text{occ}}$ of filling the cages.

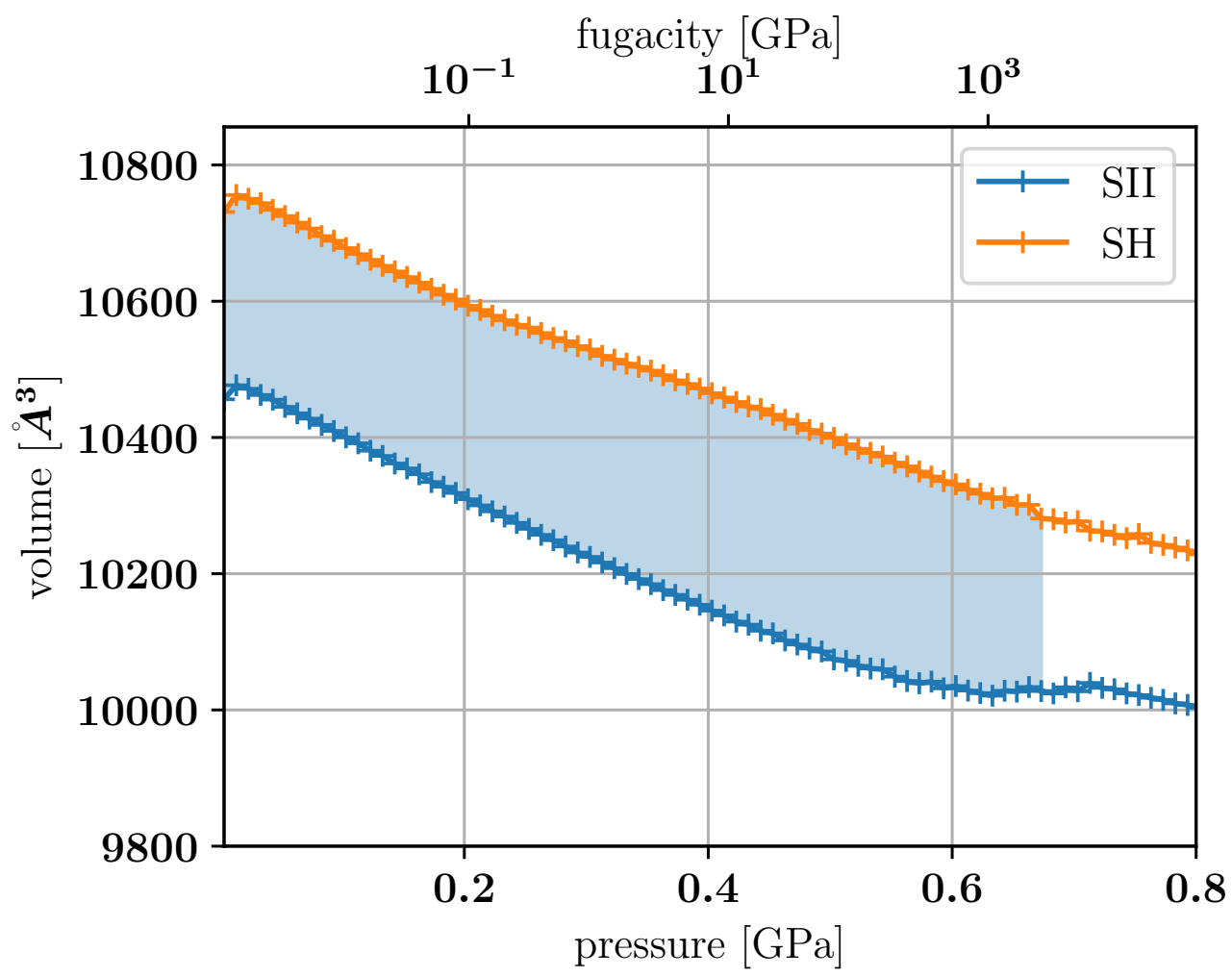


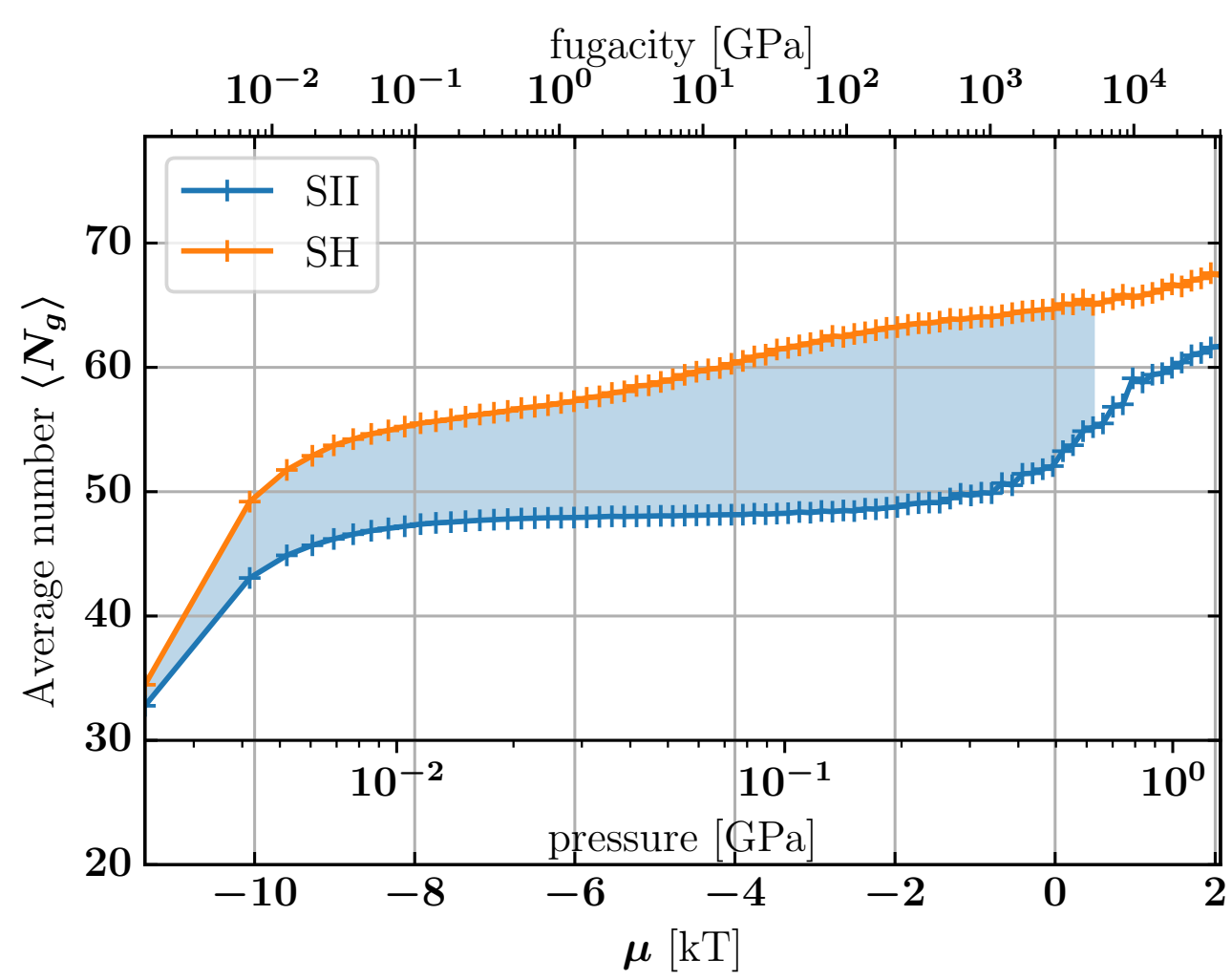


Figure S5: (Left) Volume of sII and sH methane hydrates as a function of methane pressure (at 294 K). The methane fugacity is indicated on the upper horizontal axis. (Right) absorption isotherms in sII and sH methane hydrates in terms of the methane chemical potential (corresponding pressure shown on a second horizontal axis). The shaded areas show the integral of $\langle V \rangle dP$ and $\langle N_g \rangle d\mu_g$ from 30 bar to the estimated coexistence pressure.

# 3 Spikes in the Lattice-Switch order parameter

Spikes (very short-lived large values) of the lattice-switch order parameter have been observed in our simulations of both the empty and filled clathrates. This phenomenon seems to occur more frequently in the empty clathrate. Fig. S6 shows such spikes during constant-$NPT$ simulations of an empty clathrate. The order parameter is computed for a tentative lattice-switch between structures II and H, but no such switch is actually performed.

The order parameter can attain extremely large values during spikes, reaching up to $\sim 10^{10}$ kJ/mol, whereas it typically fluctuates around $\sim 2 \times 10^6$ kJ/mol under normal conditions. In a lattice-switch simulation, each site in one structure is mapped onto a corresponding site in the other. In the present case, this mapping was initially chosen in an essentially arbitrary manner,

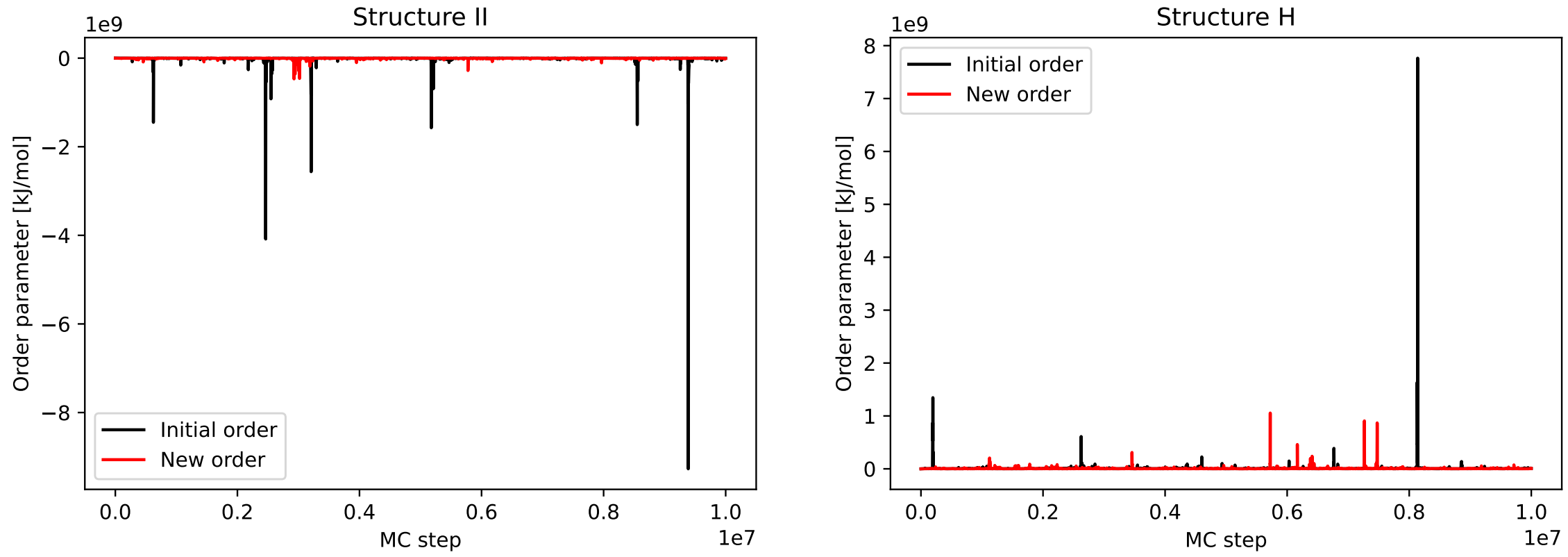


Figure S6: Lattice-Switch order parameter [eq. 23 in the main article] during an isothermal-isobaric simulation of an empty clathrate with structure II (left plot) or structure H (right plot) at 294 K and 30 bar. The curve labeled "New order" represents an alternative mapping choice between the lattice sites of structures II and H, as explained in the main text.

as described in the main article. Analysis of configurations associated with these spikes shows that they originate from pairs of molecules becoming unrealistically close under a (virtual) lattice-switch move. Although the move itself is not accepted, it is used to evaluate the energy difference. Two water molecules that are well separated in structure II may be mapped onto neighbouring lattice sites in structure H. Since these molecules interact only weakly in the original structure, their displacements relative to their reference sites are largely uncorrelated. When mapped onto adjacent sites, they can therefore overlap or come into very close proximity, generating large energy differences and hence spikes in the order parameter. This effect is particularly pronounced near the boundaries of the simulation box, where periodic boundary conditions can bring mapped neighbours into close contact. It is also more frequent in empty hydrates, where the water network is less constrained and exhibits larger displacements from lattice sites than in filled clathrates.

We attempted to construct a more physically motivated mapping between structures II and H by exploiting their shared structural features. In particular, both structures contain identical small $5^{12}$ cages. We therefore began by mapping the group of five small cages in the upper part of structure H (Fig. 2 of Ref. 1 in the bibliography of the main paper) onto the corresponding group in sII. In practice, the layer containing this group comprises seven small cages that could, in principle, be matched to sH (see Fig. S7), allowing us to identify a consistent mapping for 98 out of the 272 water molecules in the simulation box.

Further correspondences between small cages can be established up to small shifts (see the central cages in Fig. S7). However, identifying these mappings is non-trivial: the combination of rotations (Fig. S7, bottom) and periodic boundary conditions in relatively small simulation boxes introduces ambiguities. In particular, when attempting to map layers of small cages in the $xy$-plane of sH onto those in sII, some molecules expected to belong to a given layer are reassigned to neighbouring layers once periodic boundary conditions are applied to the tilted sII simulation box.

Additional water molecules in the vicinity of the small cages were mapped by following the hydrogen-bond network as closely as possible. The remaining molecules were assigned by superimposing the two simulation boxes (Fig. S7, bottom) and applying a nearest-distance criterion under periodic boundary conditions. This procedure yields a mapping between structures II and H that is somewhat more optimised, although not unique.

When using this new mapping, spikes are reduced in magnitude and occur less frequently in structure II, as illustrated by the curve labelled "new order" in Fig. S6. In structure H, the frequency of spikes appears largely unchanged, although the very large spikes observed previously are no longer present. Despite this, the improvement in the overall accuracy of the LSMC simulations is marginal. A new bias function constructed with this mapping yields a free-energy barrier to the gateway states that is essentially unchanged, indicating that the intrinsic difficulty of the simulation is unaffected. For this reason, we retained the original, essentially random, mapping between the two structures. We also note that the use of the logarithmic order parameter, Eq. 27 of main text, mitigates the impact of large excursions in the order parameter. Since the spikes are transient and contribute negligibly to the free energy, their effect on the final results is minimal.

# References


[1] J. L. F. Abascal, E. Sanz, R. García Fernández, and C. Vega. A potential model for the study of ices and amorphous water: TIP4P/Ice. *The Journal of Chemical Physics*, 122(23), 2005.

[2] Sigbjørn L. Bore, Pablo M. Piaggi, Roberto Car, and Francesco Paesani. Phase diagram of the TIP4P/Ice water model by enhanced sampling simulations. *The Journal of Chemical Physics*, 157(5), August 2022.

[3] Paul E. Brumby, Daisuke Yuhara, Tomohiro Hasegawa, David T. Wu, Amadeu K. Sum, and Kenji Yasuoka. Cage occupancies, lattice constants, and guest chemical potentials for structure II hydrogen clathrate hydrate from Gibbs ensemble Monte Carlo simulations. *The Journal of Chemical Physics*, 150(13), April 2019.

[4] Joseph Costandy, Vasileios K. Michalis, Ioannis N. Tsimpanogiannis, Athanassios K. Stubos, and Ioannis G. Economou. Molecular dynamics simulations of pure methane and carbon dioxide hydrates: lattice constants and derivative properties. *Molecular Physics*, 114(18):2672–2687, 2016.

[5] Andrey Yu. Manakov, Andrey G. Ogienko, Marek Tkacz, Janusz Lipkowski, Andrey. S. Stoporev, and Nikolay V. Kutaev. High-Pressure Gas Hydrates of Argon: Compositions and Equations of State. *The Journal of Physical Chemistry B*, 115(31):9564–9569, 2011.

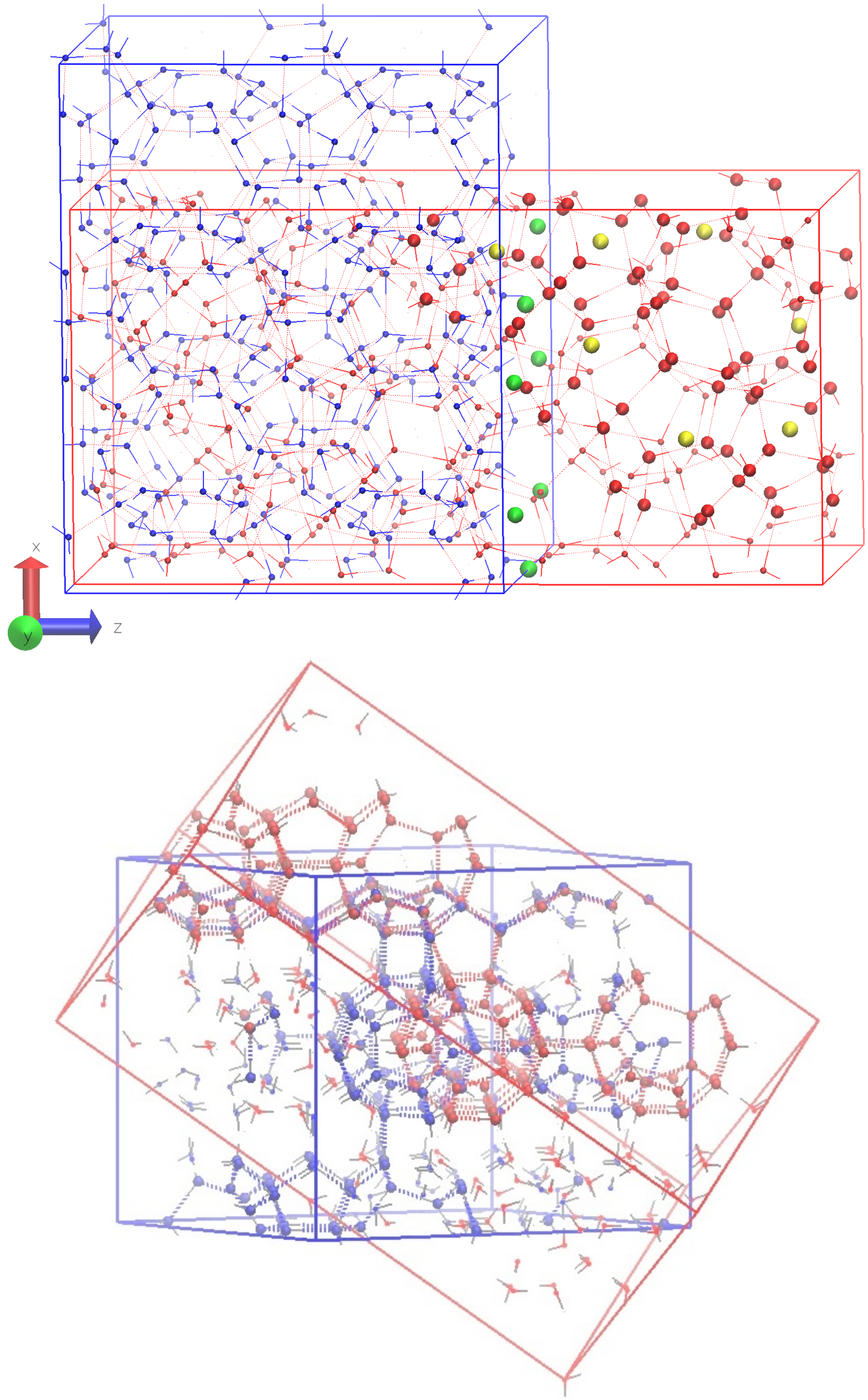


Figure S7: (top) Same as Fig. S1 but where 7 small cages in sII forming a layer are marked with larger beads and by locating their cage centre with yellow spheres. 7 green spheres mark the corresponding cage centres in sH.
(bottom) A view showing some correspondences between lattice sites in sII (rotated and shifted red box) and sH (blue box).
